# Delirium Tremens or a Deadlier Delirium? Rethinking Encephalopathy in a Patient with Chronic Alcoholism.

**DOI:** 10.51894/001c.165600

**Published:** 2026-07-31

**Authors:** Candace Fong, Matthew Zawisa, Dheeraj Thammineni

**Affiliations:** 1 McLaren - Macomb

## 82

West Nile virus (WNV) is a mosquito-borne flavivirus with most infections remaining asymptomatic; however, fewer than 1% progress to neuroinvasive disease. Risk factors for severe infection include advanced age, immunocompromise, chronic medical comorbidities, and alcohol use disorder. We present a case of WNV meningoencephalitis initially obscured by metabolic derangements and concern for alternative infectious etiologies. A 56-year-old female with heavy alcohol use presented after a generalized seizure following several days of flu-like symptoms. She was admitted with ventilator-dependent respiratory failure, shock, severe hyponatremia, and metabolic encephalopathy. Initial workup, including CT imaging, suggested no acute intracranial pathology. Lumbar puncture revealed lymphocytic pleocytosis with normal glucose and mildly elevated protein. Empiric treatment for possible bacterial and viral meningitis was initiated. Additional history obtained later revealed recent insect bites while gardening. CSF serology subsequently returned positive for WNV IgM, confirming neuroinvasive West Nile meningoencephalitis. Antibiotics were discontinued, and the patient experienced gradual neurologic improvement but retained significant weakness and cognitive deficits, requiring transfer to inpatient rehabilitation. This case highlights the importance of considering a wide variety of differentials when patients present with neurologic decline. In addition, it also demonstrates the importance of sufficient history taking. Despite the extreme rarity of the disease, neuroinvasive WNV should be considered in patients presenting with acute neurologic decline, particularly in the context of heavy alcohol use, tobacco use, and other comorbidities. Early recognition is essential to avoid unnecessary antimicrobial therapy and to guide supportive management.

